# Reward Sensitivity Enhances Ventrolateral Prefrontal Cortex Activation during Free Choice

**DOI:** 10.3389/fnins.2016.00529

**Published:** 2016-11-18

**Authors:** Catherine Cho, David V. Smith, Mauricio R. Delgado

**Affiliations:** ^1^Department of Psychology, Rutgers UniversityNewark, NJ, USA; ^2^Department of Psychology, Temple UniversityPhiladelphia, PA, USA

**Keywords:** choice, perceived control, striatum, cognitive control

## Abstract

Expressing one's preference via choice can be rewarding, particularly when decisions are voluntarily made as opposed to being forced. An open question is whether engaging in choices involving rewards recruits distinct neural systems as a function of sensitivity to reward. Reward sensitivity is a trait partly influenced by the mesolimbic dopamine system, which can impact an individual's neural and behavioral response to reward cues. Here, we investigated how reward sensitivity contributes to neural activity associated with free and forced choices. Participants underwent a simple decision-making task, which presented free- or forced-choice trials in the scanner. Each trial presented two cues (i.e., points or information) that led to monetary reward at the end of the task. In free-choice trials, participants were offered the opportunity to choose between different reward cues (e.g., points vs. information), whereas forced-choice trials forced individuals to choose within a given reward cue (e.g., information vs. information, or points vs. points). We found enhanced ventrolateral prefrontal cortex (VLPFC) activation during free choice compared to forced choice in individuals with high reward sensitivity scores. Next, using the VLPFC as a seed, we conducted a PPI analysis to identify brain regions that enhance connectivity with the VLPFC during free choice. Our PPI analyses on free vs. forced choice revealed increased VLPFC connectivity with the posterior cingulate and precentral gyrus in reward sensitive individuals. These findings suggest reward sensitivity may recruit attentional control processes during free choice potentially supporting goal-directed behavior and action selection.

## Introduction

Reward sensitivity refers to individual responsiveness to rewards and the positive affect derived from engaging in reinforcing behaviors (Gray, 1987). Sensitivity to reward can be influenced by the biological mesocorticolimbic system (Di Chiara et al., 2004; Kelley et al., 2005) and psychological traits (Cohen et al., 2005), which suggests it can vary significantly among individuals (Carver and White, 1994). Upon encountering stimuli with appetitive properties (i.e., food or drugs), reward sensitivity may predict responses to obtain such cues, reflected by heightened activations in reward-related brain regions (Volkow et al., 2002; Beaver et al., 2006; Carter et al., 2009). For instance, those with a high sensitivity to reward are more likely to experience greater cravings (Franken and Muris, 2005), recruit reward-related brain activity (Beaver et al., 2006; Hahn et al., 2009), and exhibit appetitive responses toward cues with greater reinforcement value (Volkow et al., 2002; Davis et al., 2004). These findings highlight how reinforcers tend to promote approach behavior that lead to the seeking and consumption of incentives—effects that are more pronounced in humans with greater reward sensitivity (Stephens et al., 2010).

An important way of understanding responses to reward cues is to compare decisions voluntarily made by the individual (i.e., free choice) versus predetermined choices (i.e., forced choice). Expressing one's preference via choice can be rewarding, particularly when decisions are freely made as opposed to being forced (Lieberman et al., 2001; Sharot et al., 2009, 2010). For instance, participants who were prompted to smoke on a predetermined schedule (forced choice) experienced significantly lower rewarding effects from smoking compared to those who were free to smoke (free choice) on their own schedule (Catley and Grobe, 2008). In these types of choice studies, free-choice behavior is compared to forced-choice procedure that is experimenter-determined and the resulting outcome is measured. Such studies converge on the idea that exerting control via choice enhances (a) motivation and performance (Ryan and Deci, 2000a; Patall, 2013) and (b) positive feelings and neural activity in reward-related brain regions such as the striatum when anticipating an opportunity to exert control (Leotti and Delgado, 2011, 2014). Importantly, these findings suggest having the opportunity to choose (free choice) relative to being forced to choose (forced choice) between reward options may engage distinct behavioral and neural patterns in reward sensitive individuals. Although previous work examining benefits of choice have focused on neural responses during the anticipation of choice (Sharot et al., 2009, 2010; Leotti and Delgado, 2011, 2014), the present study investigates the period of choice itself and whether engaging in choices involving rewards recruits distinct neural systems as a function of reward sensitivity.

Implementing control via choice also augments general motivation and performance (Ryan and Deci, 2000a; Patall, 2013), and prior research has suggested that the ventrolateral prefrontal cortex (VLPFC) may be sensitive to manipulations of motivation and performance (Taylor et al., 2004; Baxter et al., 2009). The VLPFC receives input from the orbitofrontal cortex and subcortical areas such as the midbrain and amygdala (Barbas and De Olmos, 1990) linked with motivational and affective information (Tremblay and Schultz, 1999; Paton et al., 2006). The VLPFC has also been associated with cognitive control processes that guide access to relevant information (Petrides et al., 1995; Duncan and Owen, 2000; Petrides, 2002; Bunge et al., 2004; Badre and Wagner, 2007) and is more activated during conditions that require goal-directed behavior (Sakagami and Pan, 2007). Interestingly, the VLPFC interacts with motor regions to orient attention (Corbetta and Shulman, 2002), suggesting increased connectivity with VLPFC might be important for directing attention to relevant stimuli in reward sensitive individuals. Altogether, these findings suggest responses to choice may vary due to individual traits such as reward sensitivity, which can be tracked by the VLPFC (Mullette-Gillman et al., 2011).

Here, we investigated how reward sensitivity contributes to neural responses associated with free and forced choices. For example, how does reward sensitivity interact with forced choices within a category (for example, broccoli and Brussels sprouts) versus free choices across categories (i.e., vegetables and snacks)? Will reward sensitive individuals demonstrate different patterns of brain activity during free relative to forced choices? In this study, we presented participants with two cues that were predictive of distinct classes of outcomes in each trial. Specifically, participants could earn points or information, both of which were tied to a monetary reward at the end of the experiment (Smith et al., 2016b). We presented these cues in two distinct formats. On free-choice trials, the cues were mixed (for example, subjects were free to choose between points or information), thus allowing participants to freely express their preference between points and information. On forced-choice trials, both cues were predictive of points or information, thus forcing the participant to choose within the given option (i.e., forced to choose within information or information), hence limiting their freedom to choose across cues. The goal of the task was to choose the option that maximized monetary reward to be obtained at the end. We focused on two key hypotheses. Based on prior studies demonstrating striatal involvement in the value of choice, we hypothesized reward sensitivity to be linked with reward signals in the striatum during free choice. Second, based on motivational control literature, we expected the VLPFC to modulate reward-related circuitry during free-choice trials in reward sensitive individuals.

## Methods

### Participants

Thirty-three healthy subjects participated in the current study (mean age = 24, range: 18–39, 18 females). Written informed consent was obtained from each subject for a protocol approved by the Institutional Review Board of Rutgers University.

### Stimuli and task

**Figure 1:**
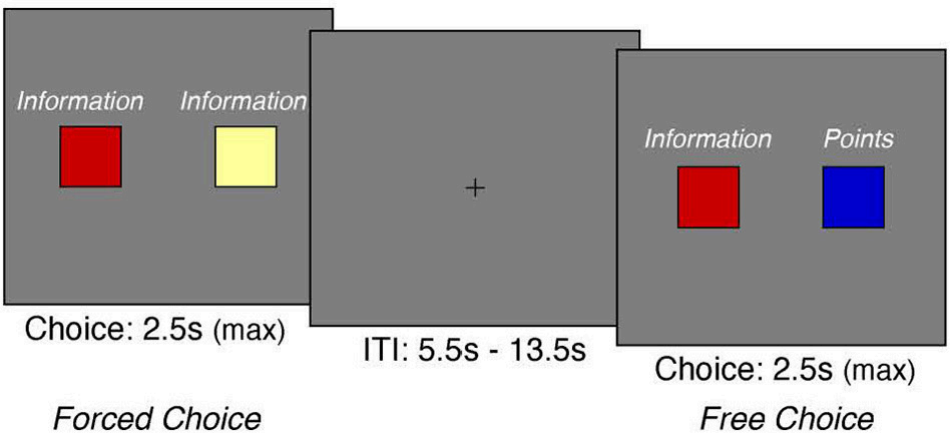
**Experimental Task Design**. On each trial, we presented participants with two cues that were predictive of points (cold colors) and/or information (warm colors)-both of which were critical for receiving monetary reward. We presented these cues in two distinct formats. During free-choice trials, the cues were mixed, thus allowing participants to freely express their preference between points and information. On forced-choice trials, both cues were predictive of points or information, thus forcing the participant to choose the given option. (Note that the words “information” and “points” were not presented to the participants during the task; they are shown here for illustrative purposes).

In an experiment prior to this task (Smith et al., 2016b), subjects performed a card task that involved learning about colors that were associated with either points or information (Figure 1). A *points trial* presented three cold colors, in which each color was associated with a point value (1, 2, or 3). An *information trial* presented three warm colors, and each color was probabilistically linked with a letter (D, K, or X). Upon selecting a color in each trial, participants received a feedback representing the value (either a point or letter) of the color. Participants performed 36 trials of each points and information trial types. Both types of trials were important because (1) subjects needed to accrue enough points to play a bonus game at the end to earn extra money, and (2) the bonus game presented a letter in each trial and subjects needed to answer correctly to win money. This task allowed participants to develop preferences for either points or information as a means to acquire reward. Further methodological details and discussion of results are described in Smith et al. (2016b).

Subsequently, on each trial of the choice task, we assessed preferences between points and information by asking participants to make a series of free and forced choices (Figure 1). On free-choice trials, participants chose freely between a cue that delivered points and a cue that delivered information. On forced-choice trials, participants were presented with two cues that delivered either points or information. This procedure blocked the participant from choosing between points and information—which effectively forced them to choose the presented option (cf. Lin et al., 2012). The goal of the task was to maximize monetary reward to be received at the end of the task. Each trial was presented for 2.5 s, during which participants could make a response. A random trial was selected at the end of the experiment to ensure that participants cared about each decision. Each trial was separated by a random intertrial interval from 5.5 to 13.5 s. At the end of the task, a randomly chosen trial from each subject's response was presented with an associated monetary reward. The present experimental task was programmed using the Psychophysics Toolbox 3 in MATLAB (Brainard, 1997; Kleiner et al., 2007).

### Temporal experience of pleasure scale (TEPS)

To probe individuals' sensitivity to rewards, we implemented the Temporal Experience of Pleasure Scale (TEPS; Gard et al., 2006). The scale is composed of two subscales measuring anticipatory (10 items) and consummatory (8 items) pleasure. Anticipatory pleasure reflects positive feelings derived from anticipation of a reinforcer, whereas consummatory pleasure measures in-the-moment feelings of joy in response to a pleasurable cues (Gard et al., 2006; Treadway and Zald, 2011). Based on previous literature which suggests perceiving control via choice is rewarding (Leotti and Delgado, 2011, 2014), we focused on the consummatory subscale as subjects were expected to increase feelings of joy upon being presented with an option of control.

### fMRI data acquisition and preprocessing

Functional magnetic resonance imaging data were acquired on a 3T Siemens MAGNETOM Trio scanner using a 12-channel head coil at the Rutgers University Brain Imaging Center (RUBIC). Whole-brain functional images were collected using a T2^*^-weighted echo-planar imaging (EPI) sequence. The parameters for the functional measurement were as follows: GRAPPA with R = 2; repetition time (TR) = 2000 ms; echo time (TE) = 30 ms; flip angle = 90°; matrix size = 68 × 68; field of view (FOV) = 204 mm; voxel size = 3.0 × 3.0 × 3.0 mm; with a total of 37 slices (10% gap). High-resolution T1-weighted structural scans were collected using a magnetization-prepared rapid gradient echo (MPRAGE) sequence (TR: 1900 ms; TE: 2.52 ms; matrix 256 × 256; FOV: 256 mm; voxel size 1.0 × 1.0 × 1.0 mm; 176 slices; flip angle: 9°). B0 field maps were also obtained following the same slice prescription and voxel dimensions as the functional images (TR: 402 ms; TE1: 7.65 ms; TE2: 5.19 ms; flip angle: 60°).

Imaging data were preprocessed using Statistical Parametric Mapping software [SPM12; Wellcome Department of Cognitive Neurology, London, UK (http://www.fil.ion.ucl.ac.uk/spm/software/spm12)]. Each image was aligned with the anterior commissure posterior commissure plane for better registration. To correct for head motion, each time series were realigned to its first volume. Using the B0 maps, we spatially unwarped each dataset to remove distortions from susceptibility artifacts. Prior to normalization of the T1 anatomical image, mean EPI image was coregistered to the anatomical scan. A unified segmentation normalization was performed on the anatomical image, which was used to reslice EPI images to MNI stereotactic space using 3-mm isotropic voxels (Ashburner and Friston, 2005). Normalized images were spatially smoothed using a 4-mm full-width-half-maximum Gaussian kernel. Additional corrections were applied to control for motion using tools from FSL (FMRIB Software Library), given that connectivity results can be particularly vulnerable to severe distortion by head motion. Motion spikes were identified by calculating the differences between the reference volume and (1) root-mean-square (RMS) intensity difference of each volume, and (2) mean RMS change in rotation/translation parameters. A boxplot threshold (i.e., 75 percentile plus 1.5 times the interquartile range) was applied to classify volumes as motion spikes. Once identified, all spikes and the extended motion parameters (i.e., squares, temporal differences, and squared temporal differences) were removed via regression (Power et al., 2015). Next, non-brain tissue was segmented and removed using robust skull stripping with the Brain Extraction Tool (BET), and the 4D dataset was globally normalized with grand mean scaling. Low frequency drift in the MR signal was removed using a high-pass temporal filter (Gaussian-weighted least-squares straight line fit, with a cutoff period of 100 s).

### fMRI analyses

Imaging data were analyzed using the FEAT (fMRI Expert Analysis Tool) module of FSL package, version 6.0. A general linear model (GLM) with local autocorrelation correction was used for our model (Woolrich et al., 2001). First, we generated a model to identify brain regions that showed increased BOLD signal as a function of free and forced choice conditions. Our GLM included five regressors to model two cues (i.e., points or information) presented to subjects during the two conditions (free and forced choice): free choice (points), forced choice (points), free choice (information), forced choice (information), and missed responses. Therefore, each condition (forced or free) consisted of two regressors (points and information). Our main contrast of interest was free > forced choice. To test our hypothesis of whether individual differences in reward sensitivity modulated distinct brain regions in response to free choice, individual scores of TEPS were entered as a covariate.

Next, we used the output of the main contrast of interest (free > forced) as a seed to conduct a psychophysiological interaction analysis (PPI; Friston et al., 1997). Recent meta-analytic work has demonstrated that PPI produces consistent and specific patterns of task-dependent brain connectivity across studies (Smith and Delgado, 2016; Smith et al., 2016a). For each individual, we extracted BOLD time-series from the peak voxel within a mask of the VLPFC cluster. The whole cluster was identified using the contrast of the regressor during free-choice vs. forced-choice trials. Next, we generated a single-subject GLM consisting of the following regressors: five main regressors/conditions, a physiological regressor representing the time course of activation within the VLPFC ROI, and interactions with VLPFC activity with each of the four main regressors. Importantly, modeling PPI effects separately for each condition (i.e., a generalized PPI model) has been shown to result in improved sensitivity and specificity (McLaren et al., 2012). We included nuisance regressors in our model to account for missed responses during the decision-making phase. All task regressors were convolved with the canonical hemodynamic response function. We modeled group-level analyses using a mixed-effects model in FLAME 1 (FMRIB's Local Analysis of Mixed Effects), treating subjects as a random effect (Beckmann et al., 2003). All z-statistic images were thresholded and corrected for multiple comparisons using an initial cluster-forming threshold of *z* > 2.3 and a corrected cluster-extent threshold of *p* <0.05 (Worsley, 2001).

## Results

In the present study, we addressed the question whether individual differences in reward sensitivity are associated with behavioral and neural responses to free and forced choice. Our behavioral analyses focused on evaluating individual difference scores in reward sensitivity with TEPS. Subsequently, we analyzed differences in reaction times in free and forced choice trials.

### Individual differences in reward sensitivity

From an experiment preceding the choice task, we found that subjects were more likely to prefer affective choices (*M* = 0.607, *SD* = 0.182) compared to informative choices (*M* = 0.393, *SD* = 0.182); *t*_(32)_ = −3.367, *p* = 0.002. They were successful at obtaining relevant information as indicated by performance in the bonus task (*M* = 69.36%; *SE* = 3.45%). The bonus task assessed the extent to which the participants learned the associations between the colors and letters, which led to a monetary bonus.

Consistent with previous research (Carter et al., 2009; Chan et al., 2012), we quantified reward sensitivity with TEPS (Gard et al., 2006). Given that our primary goal was to examine how reward sensitivity interacts with responses to free and forced choices during the decision-making phase, our subsequent analyses focused on the consummatory component of the TEPS. Individuals varied in the consummatory reward sensitivity score (TEPS-c) from 34.91 ± 4.89 (mean ± SD, ranging from 26 to 45), which was positively related with TEPS-a, *r*_(31)_ = 0.42, *p* <0.05.

Making decisions during free and forced choices might be associated with different levels of difficulty in decision making, which may yield slower or faster reaction times between conditions. To test whether subjects perceived differences in difficulty between the two trial types, we conducted a paired-sample *t*-test to measure differences in response times between free and forced-choice trials. Reaction times between free (*M* = 1.1700 s, *SD* = 0.279 s) and forced choice (*M* = 1.166 s, *SD* = 0.2697 s) were not significant [*t*_(32)_ = −0.131, *p* = 0.45]. Hence, our reaction time results suggest (1) participants perceived both types of trials as similar in difficulty, and (2) further differences in neural activity were not driven by participants' reaction times between free and forced choice trials. Nevertheless, individual differences in response times could be tied to reward sensitivity. We therefore examined whether response times for free and forced choice were correlated as a function of individual reward sensitivity (TEPS-c). We did not find a relationship between reward sensitivity scores and response times (TEPS-a and reaction time during free choice, *r* = −0.15, n.s.; TEPS-a and reaction time during forced choice, *r* = −0.08, n.s.; TEPS-c and reaction during free choice, *r* = −0.18, n.s.; and TEPS-c and reaction time during forced choice, *r* = −0.16, n.s.). Taken together, these observations suggest both types of trials did not reveal any differences in response times that could be attributed to further differences in neural activations.

### Reward sensitivity engages the VLPFC during choice

**Figure 2:**
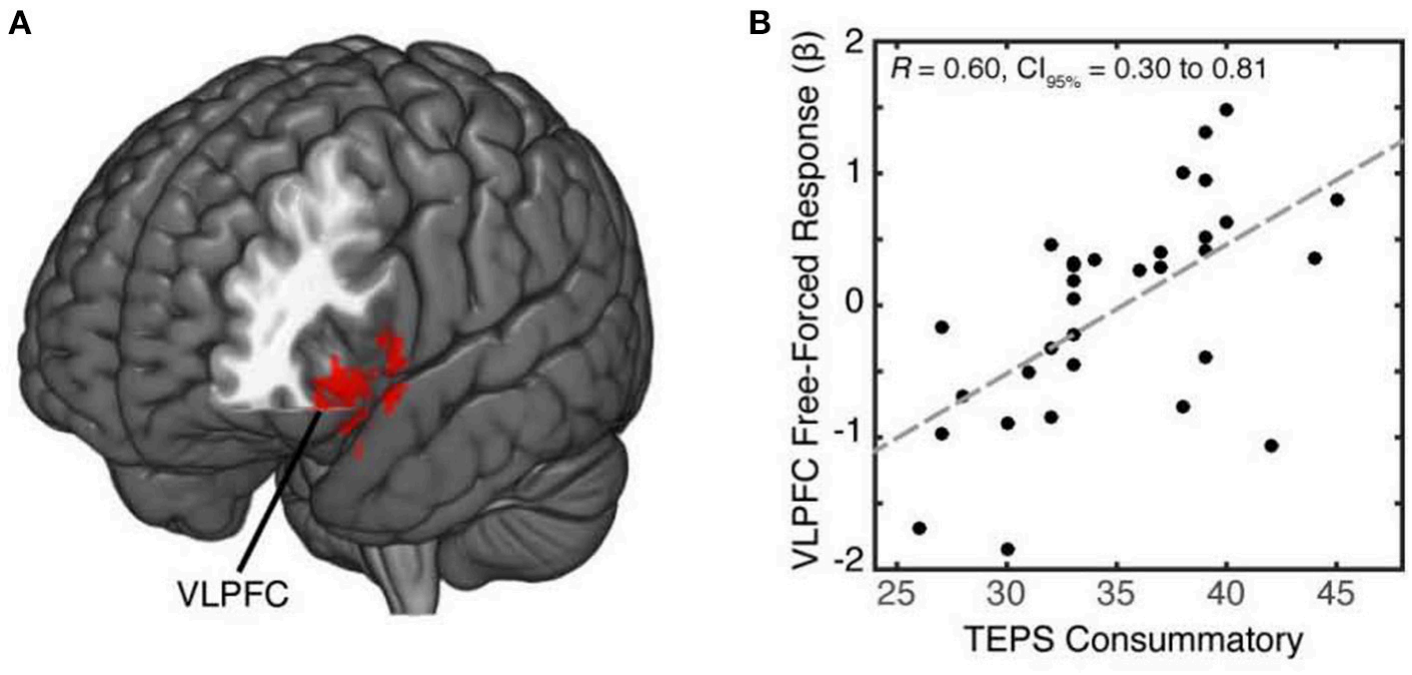
**Increased activation of VLPFC in reward sensitive individuals during free choice relative to forced choice. (A)** To identify brain regions whose activation increased during free choice (relative to forced choice) as a function of reward sensitivity, we conducted a whole-brain cluster analysis at a threshold of *z* = 2.3. We identified a cluster within the left VLPFC (MNI x,y,z = −45, 20, −22; 140 voxels) which positively correlated with individuals' reward sensitivity scores during free-relative to forced-choice trials. **(B)** We found a positive linear trend ofVLPFC activity with reward sensitivity scores, with higher activation corresponding to higher reward sensitivity and lower activation corresponding to lower reward sensitivity.

Individuals experience positive feelings when given the opportunity to freely choose (Leotti et al., 2010; Leotti and Delgado, 2011). We predicted that for reward sensitive individuals, free-choice trials would enhance reward-related neural activation modulated by VLPFC. Imaging data have found reward-motivated trials to enhance response in the VLPFC, a region previously associated with cognitive control, response selection, and reward motivation (Taylor et al., 2004; Badre and Wagner, 2007; Baxter et al., 2009). To test our hypothesis of whether individual differences in reward sensitivity modulated distinct brain regions in response to free choice, we examined neural patterns that covaried as a function of reward sensitivity during free vs. forced choice. In our whole-brain analysis of free vs. forced choice contrast, we identified a cluster within the left VLPFC (MNI x,y,z, = −48, 23, −19; 140 voxels, *p* = 0.011) (Figure 2A; Supplementary Table 1) that covaried with individuals' reward sensitivity scores (Figure 2B; see also Supplementary Figure 1). Specifically, individuals who reported greater reward sensitivity scores exhibited greater activation in the VLPFC during free choice compared to forced-choice trials. We did not observe any activations from the forced vs. free contrast that covaried with reward sensitivity scores, even at a lower uncorrected-threshold.

### Enhanced connectivity of the attentional systems and the ventrolateral prefrontal cortex during free choice

**Figure 3:**
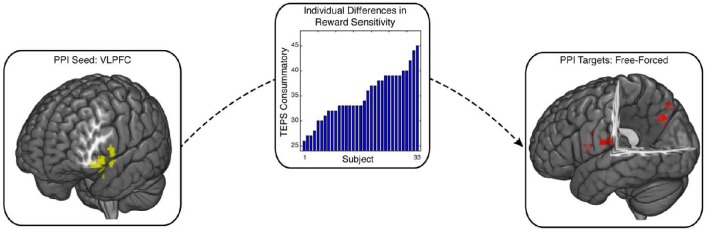
**Enhanced VLPFC connectivity with PCC and precentral gyrus during free choice compared to forced choice**. Psychophysical interaction (PPI) analysis was implemented to test whether free choice elicited greater VLPFC connectivity with other brain regions as a function of individuals' reward sensitivity scores. The seed region, (VLPFC; image on the left), exhibited enhanced connectivity with PCC and precentral gyrus (image on the right), contingent upon reward sensitivity (image in the middle). PCC is a region that has been previously associated with cognitive control, along with VLPFC. This connectivity relationship was enhanced for individuals who reported greater valuation for rewards.

Our whole-brain cluster analysis suggests a role for the VLPFC in processing free choice in reward sensitive individuals. The VLPFC is densely connected with a number of structures associated with cognitive control and motivational systems (Petrides and Pandya, 2002; Sakagami and Pan, 2007). One potential idea is that connectivity of VLPFC with these regions may support response selection and goal-directed behavior during free-choice trials. We tested this idea using a psychophysiological interaction (PPI) analysis with the VLPFC defined by the cluster analysis as our seed region (Friston et al., 1997). This analysis allowed us to identify regions whose connectivity with the VLPFC increased as a function of reward sensitivity during free relative to forced choice. Our PPI analysis identified two clusters in the posterior cingulate cortex (PCC) (MNI x,y,z = 18, −73, 41; 103 voxels, *p* = 0.043) and the precentral gyrus (MNI x,y,z = −39, −19, 35; 156 voxels, *p* = 0.0035), which showed enhanced connectivity with VLPFC as a function of individuals' reward sensitivity (Figure 3; see also Supplementary Figure 2). All coordinates are reported on Supplementary Table 1.

## Discussion

This paper investigated the influence of free choice on individual differences in reward sensitivity. When given free choice, individuals high in reward sensitivity revealed enhanced VLPFC activation, a region known to be involved in attentional control and response selection. Further, our PPI analyses found increased VLPFC connectivity with the PCC and precentral gyrus that might be involved in motor processing during free-choice trials in reward sensitive individuals. These observations suggest reward sensitivity may recruit VLPFC-related attentional control processes during free choice that relate to goal-directed behavior and action selection.

Individuals high in reward sensitivity show a tendency to engage in goal-directed behavior and to experience pleasure when exposed to cues of impending reward (Carver and White, 1994; Gard et al., 2006). When given an opportunity to freely choose between options, individuals sensitive to rewards may more readily orient to an option that leads to maximizing their goal (i.e., increasing chances of reward). The VLPFC has been associated with computing behavioral significance by integrating input from regions processing motivational and affective information (Sakagami and Pan, 2007). Experiencing control via exerting self-initiated choice is motivating and can be rewarding in it of itself (Ryan and Deci, 2000b; Bhanji and Delgado, 2014; Leotti et al., 2015). A region linked with tracking reward expectancy value (Pochon et al., 2002), the recruitment of VLPFC during free choice in reward sensitive individuals might suggest a role for the region in increasing attentional control (Duncan and Owen, 2000; Bunge et al., 2004; Badre and Wagner, 2007) and guiding goal-directed behavior (Sakagami and Pan, 2007). Consistent with this idea, greater VLPFC activation is observed when individuals make decisions between consequential choices (i.e., choosing with whom to date) relative to inconsequential choices (i.e., choosing between same-sex faces) (Turk et al., 2004). Our results suggest that an opportunity for free choice enhances attentional biases to maximize goals (i.e., reward maximization) by increasing VLPFC activation in reward sensitive individuals. Exerting control by expressing one's choice has been linked with adaptive consequences (Bandura, 1997; Ryan and Deci, 2006; Leotti et al., 2010), and one's ability to choose between alternatives modulates expectancy toward hedonic and/or aversive outcomes (Sharot et al., 2009, 2010; Leotti and Delgado, 2014). Our findings may extend to interpretations of motivational influences of free choice on reward sensitivity by modulating cognitive control regions that may facilitate goal-directed behavior.

A novel aspect of our study was that when individuals chose freely between options, we observed increased VLPFC connectivity with the precentral gyrus and the PCC/precuneus. Although the PCC is commonly associated with the default-mode network (Buckner et al., 2008), recent findings have suggested that the PCC can also participate in the cognitive control network (Leech et al., 2011, 2012; Utevsky et al., 2014). This observation has led some researchers to argue that the PCC is involved in detecting and responding to stimuli that demand behavioral modifications (Pearson et al., 2011; Leech and Sharp, 2014). By interacting with regions involved in cognitive control, the PCC might help individuals more readily orient to options during free choice and increase efficacy of behavioral responses promoting maximization of one's goals (i.e., earning rewards). In line with this explanation, our results indicate making a choice between two attributes is facilitated by increased VLPFC connectivity with the PCC. This finding yields a potential interpretation that free choice enhances VLPFC region connectivity with target PCC and precentral regions to support motor responses in reward sensitive individuals. A recent meta-analysis of PPI studies found PCC was a reliable target of studies examining cognitive control, but only when the dorsolateral prefrontal cortex was used as the seed region (Smith et al., 2016a). This discrepancy could be due to the fact that our results are based on individual differences in reward sensitivity, which were explicitly ignored in the recent PPI meta-analysis. In addition, because our analyses are limited to connectivity between regions and not directionality, an alternative explanation accounts for VLPFC region modulating PCC in response to context-specific factors (i.e., free choice) modulated by individual differences in reward sensitivity. The brain engages in multiple processes of valuation when deciding between different options. Once the sensory information is computed, signals are integrated with other motivational and contextual factors, which are then used to guide choices (Kable and Glimcher, 2009; Grabenhorst and Rolls, 2011). The interactions between the VLPFC and PCC increased during free choice, suggesting cognitive resources are made accessible via enhanced connectivity of the PCC with the cognitive control network (Leech et al., 2012; Utevsky et al., 2014).

Contrary to our hypothesis, we did not observe an association between reward sensitivity and reward signals in the striatum during free choice. Studies suggest striatal recruitment can depend on variables such as individual differences (i.e., preference for choice) and can be influenced by contextual factors (such as gain or loss context) (Leotti and Delgado, 2014). It is possible that individuals value opportunity for choice because they believe such choice will provide them access to the best option available. Consistent with this idea, previous studies found reward-related striatal activation is limited to free-choice biases primarily predicting positive outcomes (Leotti and Delgado, 2011, 2014; Cockburn et al., 2014). Hence, it is possible that the striatum did not dissociate between free and forced choices because value across both conditions were similar. It is also noteworthy that we did not observe neural activations involved in value computation in our contrasts that correlated with reward sensitivity scores. In our experimental design, there were no differences in value representation between points and information because they both led to monetary rewards. Therefore, a potential explanation for not finding value modulated brain areas as a function of reward sensitivity might account for similarity of value associated with each option.

The purpose of the current study was to compare free vs. forced choices involving rewards not necessarily prescribed to negative or positive outcomes, and both types of choice trials were related to accomplishing the goal of the task (i.e., accruing monetary outcomes). The opportunity to choose involves making a decision by selecting a response necessary for obtaining one's goals, processes supported by VLPFC (Duncan and Owen, 2000; Bunge et al., 2004; Badre and Wagner, 2007; Sakagami and Pan, 2007). Consistent with this perspective, the VLPFC emerged as a key region in our paradigm that tracked the opportunity for choice in reward sensitive individuals. In sum, our results suggest goal-directed behavior (i.e., increasing chances of reward) might be facilitated in reward sensitive individuals by enhanced attentional and cognitive control network in response to trials that afford them free choice.

We note that our reward sensitivity findings may have important clinical implications. Psychiatric patients often show deficits in decision-making tasks involving rewards (Parvaz et al., 2012). For instance, substance abuse and psychopathy are related to high levels of responsiveness to rewards (Buckholtz et al., 2010; Schneider et al., 2012; Yau et al., 2012; Tanabe et al., 2013). Reward sensitivity is also associated with negative mental-health outcomes such as greater risks for addictions (Kreek et al., 2005), alcohol abuse (Franken, 2002), eating disorders (Davis et al., 2004; Franken and Muris, 2005), and depressive disorders (Alloy et al., 2016). Consistent with our findings from reward sensitivity, bipolar patients show hypersensitivity to rewards and recruit VLPFC when anticipating rewards (Whitton et al., 2015; Chase et al., 2016), and pathological gamblers tend to reveal hyposensitivity to rewards and fail to activate the VLPFC in response to monetary rewards (de Ruiter et al., 2009). The close association between levels of sensitivity for rewards and psychiatric disorders may implicate failures in the executive control network during affective and motivational processing (Johnstone et al., 2007; Heatherton and Wagner, 2011). An exciting direction for future research is with respect to understanding networks and the specific connectivity affected by reward in/sensitivity that govern decision-making processes.

Although our results may have implications for clinical research, we note that a number of limitations accompany our results. First, in our design, free choice offered individuals a choice between dissimilar options, whereas forced-choice trials provided participants with similar options. It is possible that subjects perceived forced-choice trials as easier than choice trials upon making decisions. However, analyses on reaction times did not reveal significant differences between free- vs. forced-choice trials, which suggest our findings were not due to differences in perceived difficulty between trial types. Also, an alternative approach to human behavior of preferring forced choice could be explained by the regret theory (Loomes and Sugden, 1982), which suggests feelings of regret are enhanced when the option taken leads to a worse outcome than the alternative option. Therefore, humans may have a tendency for a status quo bias to minimize a feeling of regret (Nicolle et al., 2011). Moreover, although some aspects of our design did not measure forced choice explicitly in line with conventional free vs. forced choice framework, given that reward sensitive regions are context specific and varies in accordance with the range of possible options available (Nieuwenhuis et al., 2005), the current study tested whether brain activations in reward sensitivity would dissociate responses to free choices between categories compared with forced choices within a category. Second, it is also worth noting that the VLPFC has been implicated in a number of roles including incentive motivation (Taylor et al., 2004; Baxter et al., 2009), cognitive regulation (Lopez et al., 2014), response inhibition (Aron et al., 2004), and task switching (Braver et al., 2003). Therefore, there may be alternative explanations for the VLPFC activation in reward sensitive individuals. However, research demonstrates consistency of VLPFC activation patterns in response to rewarding stimuli as a function of reward sensitivity (de Ruiter et al., 2009; Yokum et al., 2011; Whitton et al., 2015), suggesting a role for the region in modulating attentional control in response to rewarding contexts particularly for reward sensitive individuals. Finally, we note that complex personality traits such as reward sensitivity can be difficult to quantify. Although TEPS provides one validated approach for measuring reward sensitivity, we note that other scales have also been used to relate brain responses to personality traits associated with reward and motivation. For instance, recent work has demonstrated that individual differences in regulatory focus are associated with PCC responses to promotion goals (Strauman et al., 2013) and ventral striatal responses to reward (Scult et al., 2016). These observations suggest that future work may be able to build on our findings be integrating regulatory focus theory and other personality measures with classical measures of reward sensitivity (e.g., TEPS and BIS/BAS).

Despite these caveats, our findings suggest that reward sensitivity may be an important factor in determining one's responses to rewarding stimuli. This can extend to intrinsically rewarding stimuli, such as the opportunity to exert control. When given an opportunity to make a free choice, high reward sensitive individuals might demonstrate enhanced reactivity and engage greater attentional and motor control necessary for achieving one's goals (i.e., accruing more money).

## Author contributions

CC, DS, and MD designed the research; CC and DS performed research; CC and DS analyzed data; CC, DS, and MD wrote the manuscript.

### Conflict of interest statement

The authors declare that the research was conducted in the absence of any commercial or financial relationships that could be construed as a potential conflict of interest.
