# Supplementary material for: Reward Sensitivity Enhances Ventrolateral Prefrontal Cortex Activation during Free Choice
Source: Front Neurosci. 2016 Nov 18;10:529. doi: 10.3389/fnins.2016.00529 (PMC5114280; doi:10.3389/fnins.2016.00529)
Supplement: Supplementary file 1 [file Table1.PDF]

Table 1.

(A) Neural activations that covary with reward sensitivity scores

Region	*x*	*y*	*z*	*p*-value	*k*
Free>Forced					
Ventrolateral prefrontal cortex	-48	23	-19	0.011	140

(B) PPI analysis with left VLPFC seed as a function of reward sensitivity

Region	*x*	*y*	*z*	*p*-value	*k*
Free>Forced					
Posterior cingulate cortex/precuneus	18	-73	41	0.043	103
Precentral gyrus	-39	-19	35	0.0035	156

(A) A whole-brain analysis results showing significant activation at a corrected cluster-extent threshold of $p < 0.05$, *x*,*y*, and *z* are MNI coordinates, *k* is number of significant voxels. We did not find any significant activations in the forced>free choice contrast. (B) Regions showing left VLPFC coupling at a corrected cluster-extent threshold of p < 0.05, *x*,*y*, and *z* are MNI coordinates, *k* is number of significant voxels. We did not find any significant activations in the forced>free choice contrast.
