# Supplementary material for: Reward Sensitivity Enhances Ventrolateral Prefrontal Cortex Activation during Free Choice
Source: Front Neurosci. 2016 Nov 18;10:529. doi: 10.3389/fnins.2016.00529 (PMC5114280; doi:10.3389/fnins.2016.00529)
Supplement: Supplementary file 2 [file Image1.pdf]

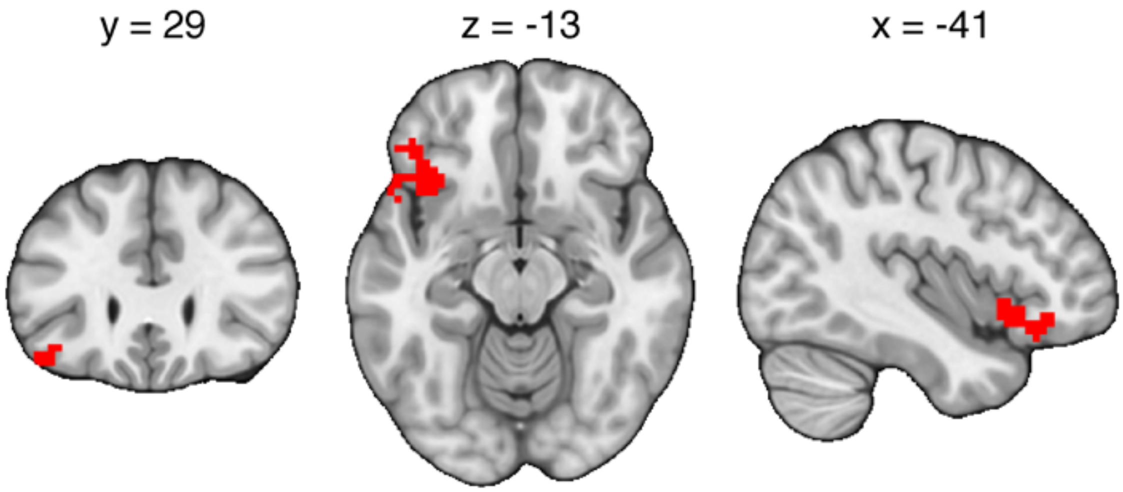

Supplementary figure 1. Slice images of activation patterns that show a positive relationship with reward sensitivity during free choice
