# Supplementary material for: Reward Sensitivity Enhances Ventrolateral Prefrontal Cortex Activation during Free Choice
Source: Front Neurosci. 2016 Nov 18;10:529. doi: 10.3389/fnins.2016.00529 (PMC5114280; doi:10.3389/fnins.2016.00529)
Supplement: Supplementary file 3 [file Image2.pdf]

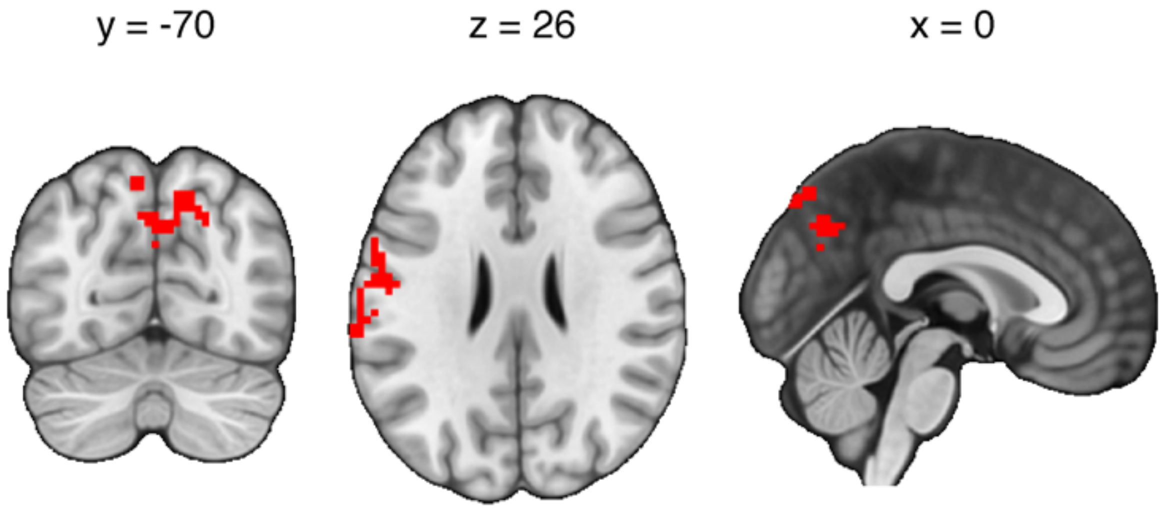

Supplementary figure 2. Slice images of activation maps resulting from psychophysiological interaction (PPI) analysis with ventrolateral prefrontal cortex seed as a function of reward sensitivity
